# Liquid biopsy: a step forward in laboratory medicine

**DOI:** 10.1515/almed-2025-0116

**Published:** 2025-09-08

**Authors:** Angel Díaz-Lagares, Wladimiro Jiménez

**Affiliations:** Epigenomics Unit, Cancer Epigenomics, Translational Medical Oncology Group (ONCOMET), Health Research Institute of Santiago de Compostela (IDIS), University Clinical Hospital of Santiago (CHUS/SERGAS), Santiago de Compostela, Spain; Centro de Investigación Biomédica en Red Cáncer (CIBERONC), ISCIII, Madrid, Spain; Department of Clinical Analysis, University Hospital Complex of Santiago de Compostela (CHUS), Santiago de Compostela, Spain; Servicio Bioqu í mica i Gen. Mol.Catedr á tico Bioqu í mica, Hospital Clinic / Fac. Medicina, Universitat Barcelona, Barcelona, Spain

**Keywords:** liquid biopsy, precision medicine, ctDNA, cfDNA, CTC, extracellular vesicle

The current issue of *Advances in Laboratory Medicine* focuses on liquid biopsy, a non-invasive diagnostic method that is going to transform the routine practice of our clinical laboratories. Liquid biopsy analyzes circulating tumor DNA (ctDNA), cell-free DNA (cfDNA), circulating tumor cells (CTCs) or extracellular vesicles (e.g., exosomes) found in body fluids such as blood, urine or cerebrospinal fluid. Liquid biopsy offers a safer, reproducible and dynamic approach to prenatal monitoring of fetal aneuploidies and other abnormalities of pregnancy, as well as for tracking tumor evolution, treatment response or resistance mechanisms [1]. This non-invasive approach was first to be employed in the 1990s for the detection of tumor-associated mutations in ctDNA. However, it was not until 2010 that the term liquid biopsy was formally introduced [2].

Next, during the first decade of this century, the development of high-throughput and sensitive techniques, such as digital PCR and next generation sequencing (NGS), envisioned a myriad of opportunities for liquid biopsy. The first liquid biopsy test (Roche Cobas EGFR Mutation test) for detecting EGFR mutations in non-small cell lung cancer was approved by the US Federal Drug Administration (FDA) in 2016. This was a major milestone that established the clinical utility of liquid biopsy in diagnostic oncology. Several technological advancements have been driving the adoption of liquid biopsy in clinical laboratories. First, the development of NGS techniques has enabled the sequencing of millions of DNA fragments, allowing the detection of low-frequency mutations in ctDNA. Second, digital PCR provides highly sensitive detection of specific mutations and enables precise quantification of rare mutant alleles. Third, microfluidic platforms have significantly improved the isolation and analysis of rare CTCs from blood samples. Fourth, exosomes – small extracellular vesicles secreted by tumor cells that contain DNA, RNA, and proteins – represent another reliable source of tumor biomarkers. Finally, advances in bioinformatics and artificial intelligence (AI) have enhanced the interpretation of complex genomic data.

Liquid biopsy offers several advantages that support its clinical application. This non-invasive approach eliminates the need for surgical procedures, reducing patient discomfort and procedural risks. It is particularly valuable in patients with advanced disease, poor performance status, or tumors in inaccessible locations. This ease of sampling also facilitates repeated testing over time, which is impractical with tissue biopsies. In addition, unlike a tissue biopsy, which provides a snapshot of a single tumor site at one time point, liquid biopsy can reflect spatial and temporal tumor heterogeneity. The analytes released by tumor cells into the bloodstream originate from multiple tumor sites, which enables to obtain a more comprehensive molecular portrait of the disease [3].

The most established clinical application of liquid biopsy is in oncology, in which the analyses of ctDNA enables the detection of somatic mutations, gene fusions, epigenetic changes, and copy number variations, offering a dynamic representation of tumor burden and molecular heterogeneity. In clinical practice, the analysis of ctDNA has demonstrated utility in several scenarios (especially when tissue is limited or inaccessible), including early detection of cancer, selection of targeted therapies, detection of resistance mutations to provide subsequent lines of therapy, monitoring treatment response and minimal residual disease (MRD) or early relapse detection, often preceding imaging-based findings. Of note, this clinical utility has been demonstrated in a wide range of malignancies, including lung, breast, colorectal, pancreatic, and prostate cancers, among others. Furthermore, multicancer panels and tumor-agnostic approaches are being developed for comprehensive genomic and epigenomic profiling using cfDNA [4].

One of the transformative strengths of liquid biopsy lies in its ability to facilitate personalized treatment. By capturing tumor evolution and clonal dynamics, it allows clinicians to tailor therapies based on real-time molecular data. This dynamic monitoring is crucial in settings such as immunotherapy, in which early identification of non-responders or cases with hyperprogression can guide timely adjustments in treatment strategy. Liquid biopsy also enables longitudinal disease surveillance, which is critical for detecting MRD or early recurrence in patients in remission. In this context, liquid biopsy serves as a molecular sentinel, often detecting relapse weeks or months before imaging, allowing for earlier intervention [2].

However, liquid biopsy is not limited to cancer. In prenatal medicine, cfDNA from maternal plasma is now widely used for non-invasive prenatal testing (NIPT), enabling the detection of fetal aneuploidies with high accuracy and minimal risk. In transplantation, donor-derived cfDNA (dd-cfDNA) serves as an early marker of organ rejection, providing valuable information before clinical deterioration or histological changes are evident. In infectious diseases, cfDNA sequencing allows pathogen identification directly from plasma, which is particularly useful in critically ill patients or when cultures are negative. Additionally, ongoing research is evaluating the role of cfDNA and other liquid biopsy analytes in autoimmune diseases, cardiovascular disease, endocrinological alterations and neurodegenerative disorders, in which cell injury and turnover may leave molecular footprints in the circulation.

Despite this promising landscape, liquid biopsy still faces important technical and clinical challenges. Among them, sensitivity is currently one of the major limitations. In fact, the low abundance of ctDNA in early-stage cancers or tumors with reduced shedding rates represents a significant complication in obtaining reliable results. In these cases, ctDNA may constitute less than 0.01 % of cfDNA, making detection extremely challenging. This may result in false negatives, especially in early detection or MRD monitoring. A lack of specificity can also occur. For instance, false positives may arise under conditions such as clonal hematopoiesis of indeterminate potential (CHIP). In these cases, blood cells acquire somatic mutations unrelated to cancer that can be misinterpreted as tumor-derived, leading to incorrect decisions. Moreover, special mention should be given to the absence of universal standardization for sample collection, processing, and analysis in liquid biopsy. Variability in pre-analytical and analytical procedures can affect reproducibility and add further difficulties when comparing the results of different laboratories [5].

Despite technological advances, the cost of NGS-based liquid biopsy assays remains high, which may limit access in certain healthcare settings. Reimbursement policies, regulatory approvals, and clinical guidelines are still evolving, creating variability in test adoption across countries and institutions. In this context, the standardization of liquid biopsy procedures and adherence to external quality assurance programs are crucial to ensure best practices, reliable results, and clinical trust. In addition, the number of liquid biopsy assays that have undergone rigorous clinical validation is still limited. Therefore, prospective, large-scale studies are needed to establish their utility across different types and stages of cancer.

The field of liquid biopsy is undergoing rapid and significant growth. Emerging technologies such as omic approaches (e,g. epigenomics and fragmentomics), machine learning algorithms, and multi-analyte assays promise to enhance diagnostic performance and expand clinical applications. For laboratory medicine, liquid biopsy represents a unique opportunity to lead innovation at the interface of molecular diagnostics and clinical care. By adopting this technology, clinical laboratories can contribute to more timely diagnoses, better patient outcomes, and more personalized healthcare. In summary, liquid biopsy is not only a technological innovation but also a transformative approach that is transforming how we understand and manage disease. Its implementation in routine laboratory practice, while still facing obstacles, is an essential step forward in the evolution of precision medicine.
